# Asynchronous Delivery of a 400 Level, Partially Peer-Graded, Oral Presentation and Discussion Course in Systems Neuroscience for 60 Students during the COVID-19 Pandemic

**DOI:** 10.3390/brainsci11060693

**Published:** 2021-05-25

**Authors:** Jack Moffat, Charlotte Copas, Kate Wood, J. David Spafford

**Affiliations:** 1 Department of Biology, University of Waterloo, Waterloo, ON N2L 3G1, Canada; jack.moffat@uwaterloo.ca (J.M.); ccopas@uwaterloo.ca (C.C.); 2 IT Professional Development Advisory Group, Science Computing Helpdesk, University of Waterloo, Waterloo, ON N2L 3G1, Canada; kate.wood@uwaterloo.ca

**Keywords:** asynchronous delivery, BONGO Video Assignments, Python Programming Scripts, remote online learning, virtual classroom, narrated PowerPoint MP4 videos

## Abstract

A 400-level undergraduate oral presentation and discussion course in Systems Neuroscience was delivered asynchronously online during the COVID-19 pandemic. Enrolled students banked their narrated oral presentations in video format online then engaged in peer evaluation in *virtual classrooms* through the course website. Student delivered their oral presentation and responded to peer questions at their leisure and convenience, without the stress and anxiety associated with a “live” performance delivery in front of their peers. A remote and asynchronously delivered course facilitated much more peer contact than “live” versions of the course, which included a total of 62 uploaded presentations, 301 video responses uploaded to 1985 questions posed by peers, a total of 1159 feedback questionnaires submitted, 1066 rankings submitted of viewed oral presentations, and 1091 scores submitted evaluating the quality of questions posed by reviewers of oral presentations. A major drawback in the remote, asynchronous deliver was the enormity of peer engagement through the course website portal, which was mostly blind to the instructor because of the inability to effectively cross-index data linked between the student entries in the LEARN course website and the uploaded videos stored within BONGO Video Assignment tool. Nonetheless, a consistent engagement of students, and the positive feedback from enrolled students, indicate that a future version of this oral/written discussion course will be delivered, in part, remotely and asynchronously, even without a mandated delivery of the course by a remote and asynchronous method due to the COVID-19 pandemic restrictions in 2020–2021.

## Highlights

- isolation and lack of learning opportunities with peers during the COVID-19 pandemic
- course centered on peer communication in remote, asynchronous learning environment
- online “How-To” videos guided students in 400 level undergraduate course
- communication between oral presenter and 4 questioners in 244 virtual classrooms
- self-grading from 1066 peer rankings of 363 video uploads, 1985 posted questions

## 1. Introduction

By Spring 2020, it was evident that an in-person, 400 level undergraduate, communication course in Systems Neuroscience in Fall 2020 would have to be adapted for remote, asynchronous delivery in an online environment to enrolled students because of the COVID-19 pandemic. Challenges of the COVID-19 pandemic in 2020 and 2021 to teaching [1,2,3], including the undergraduate medical [4,5,6,7] and neuroscience curricula [8,9], have been documented by many. Online learning experiences are often solitary learning experiences consisting of following textbook reading assignments, and downloading and watching lecture videos. Student learning is usually assessed through completion of online quizzes/exams. 

Forced social isolation and lack of a daily structure of in-person attendance in-person classes during the COVID-19 pandemic, deprive students of regular peer contact and learning opportunities from their peers. 

What we present here is how we created an online course which was structured to facilitate regular peer engagement and evaluation, in spite of the remote and asynchronous learning environment due to the COVID-19 pandemic, and working with a ballooned enrollment of ~sixty students.

Here we discuss the granular details of how a senior, 400-level oral presentation and discussion course was ported to an online environment, with delivery of oral presentations and response to questions amongst 60 enrolled students, using LEARN (D2L) course website portal and BONGO Video Assignment tool. We were able to deliver an accessible, mostly self-guided, asynchronous oral presentation and discussion course, amongst 60 enrolled students. We discuss both positive and negative issues related to working with the BONGO Video Assignment tool. A major issue we encountered is the lack of integration, and ability to cross-search, and easily collect data between uploaded/entered information by students using the BONGO Video Assignment tool and what was entered by students using available tools within the LEARN (D2L) course website. Macro scripts were programmed to automatically log in and harvest data from the LEARN (D2L) course website so each student could receive a weekly update of ongoing peer evaluation results by quizzes entered by oral presenters and questioners of oral presenters.

Overall, student engagement during the course was high, based on the consistent student participation in fulfilling required tasks during the course. Voluntary student feedback on the course was limited, but overall suggested that the course was a positive, unique and important learning opportunity for them.

While it does not replace the learning gained in “*live*” face-to-face classroom discussions, it is possible to facilitate meaningful and regular peer engagement and learning within a remote, flexible, asynchronous learning environment, to potentially hundreds of enrolled students, run by only a single lecturer and a couple of teaching assistants.

## 2. Material and Method

### 2.1. Online Remote Learning Tools

A course website portal was created within LEARN (Desire2Learn, Kitchener, ON, Canada), containing embedded Video Assignment tool, BONGO (BONGO, Loveland, CO, Canada). Weekly synchronous student help sessions were communicated by video conferencing using Microsoft Teams.

### 2.2. Course Schedule

**PART A**: Each enrolled student chooses one unique research topic out of 180 available research topics within the first week (Appendix A—Listing of available research topics and instructions in how to choose a research topic)

**PART B**: Enrolled students then have five more weeks to research their chosen research topic (Appendix B—Instructions in how to research your research topic (over weeks and weeks of preparation) and then:

- post a referenced “Nature-style” scientific abstract (must be < 750 words) as a “New Discussion Thread” within LEARN course website and append associated PDF documents of 1 or 2 review articles, and 3 to 5 scientific research articles containing experiments, pertaining to their abstract and oral presentation to their Discussion Thread (Appendix C—Instructions in how to write your research abstract; Appendix D—Instructions in how to post your research abstract).
- upload a MP4-formatted narrated PowerPoint oral presentation on their research topic within BONGO Video Assignment Tool (Appendix E—Instructions in how to create a narrated oral presentation video in MP4 format).

**PART C**: Each student evaluates and responds to questions of four oral presentations they view per week, carrying out tasks in the following order (2.3.1 to 2.3.3, below), repeated over five weeks.

- Each student has one week to view four oral presentations within their Video Assignment Folder (provided randomly from the pool of all oral presentations in BONGO) and to submit at least one *thoughtful and unique* question as “Reply” to “Thread” under the research abstract entry of each of the four oral presentations assigned to each student. (Appendix F—Instructions in how to prepare and ask at least one *thoughtful and unique* question in a “Reply” to “Discussion Thread”).
- Within three days henceforth, the oral presenter is required to post a video response addressing the questions posed by the four peers who viewed the oral presentation and posted questions under the “Reply” to “Thread” (Appendix G—Instructions in how to find your questions posted of your oral presentation and how to create and upload a question response video).
- Within three days henceforth, both oral presenters and questioners submit evaluations of each other:
- Evaluation of questioners: Oral presenters grade each questioners of their presentation that week on whether they had been asked *at least one thoughtful and unique* question in response to viewing their oral presentation. (Appendix H—Instructions in how to measure and evaluate the quality of questions of your oral presentation from questioners).
- Evaluation of oral presenters: Each of the four reviewers/questioners of oral presenters rate the quality of the oral presentation materials, the quality of oral presentation delivery, and the ability of the oral presenter to respond to questions (on a 1 to 5 scale). There is also mandatory entry of constructive feedback submitted to the oral presenters which they viewed that week (Appendix I—Instructions in how to evaluate oral presenters by feedback questionnaire rubric). In addition, all reviewers/questioners rank the four oral presentation which they viewed that week in order of: *Best*, *Second Best*, *Third Best*, and *Least Favorite*. (Appendix J—Instructions in how to rank the four oral presentations that you have watched and assessed during the week).

**PART D**: During the final exam period, students submit a final research report based on their submitted abstract and oral presentation topic (Appendix K—Instructions in how to draft your final written submissions (serving *in lieu* of a final exam) and how they will be assessed) and submit a standard research essay on a unique systems neuroscience topic not chosen as oral presentation topics by students, assigned to them within the first six weeks of the course (out of the pool of 180 available research topics, Appendix A—Listing of available research topics and instructions in how to choose a research topic).

### 2.3. Student Guidance

- A set of 10 upload How-to videos to the LEARN (D2L) course website provided students with visual instructions on how to carry out the required tasks during the course:

Video #1: How to choose a research topic (1 min, 29 s)

Video #2: How to research your research topic (over weeks and weeks of preparation) (25 min, 28 s)

Video #3: How to write and post your abstract on your research topic (25 min, 43 s)

Video #4: Instructions for writing your written submission and how they will be assessed (14 min, 39 s)

Video #5: How to generate, finalize and upload your oral presentation (8 min, 52s)

Video #6: How to prepare and ask a question or two after watching an oral presentation (3 min, 1 s)

Video #7: How to find your questions of your oral presentation and how to create and upload a question response video (8 min, 15 s)

Video #8: How to measure and evaluate the quality of questions of your oral presentation from questioners (3 min, 47 s)

Video #9: How to fill feedback questionnaire rubric of oral presentations (2 min, 38 s)

Video #10: How to rank the four oral presentations that you have watched and assessed during the week. (1 min, 29 s)

- Sample written abstracts, research reports, and videos on how to create oral presentations and video responses to peer questions were created by the teaching assistants or previous enrolled students in the course (*with their copyright permission*) were available for downloading and viewing by enrolled students.
- Regular, usually daily mass emails were sent out to students during the course providing guidance and reminders of impeding deadlines.

### 2.4. Peer Evaluation Entries Were Automatically Mined from the LEARN (D2L) Course Website, and Sent as Email Reports to Each Student Using Python Scripting Language

Over a five-week period in the latter half (Part B) of the course, each student posted questions of the four oral presentations that they viewed over the week under the appropriate research topic Discussion Thread, and then each oral presenter uploaded video responses to the questions posed by four different students under their research topic Discussion Thread. By the end of the week, the oral presenters and the questioners of the oral presenters were to evaluate each other by online quiz questionnaire. 

We ensured that all 60 students received individual emails every week as a student’s notebook of their collated peer evaluation feedback by coding in Python, a scripting language, with instructions to automatically log in and collect reports generated in the LEARN (D2L) course website (requestReports.py) and then download these reports of student data entries contained in the LEARN (D2L) course website (downloadReports.py). Different scripts in Python were coded to automatically assemble the peer evaluation reports downloaded from the LEARN (D2L) course website into spreadsheets to generate a weekly feedback report (generateWeeklyReports.ipynb), updated as a student’s notebook of aggregate peer reviews over the many weeks of peer evaluation (MarkingNotebook.ipynb). Then this peer review feedback report was automatically appended to individual emails and sent to each student (send Emails.py). We provided diagrammatic representations (flowcharts) of the Python script sequences and associated folder structures used to data mine the Learn (D2L) course website for student peer review entries and collated the data entries into individual student notebooks for sending to each student by email (Appendix L—Flowcharts of Python programming script sequences and folder structure for automatically sending weekly peer review feedback reports to enrolled students). In the Supplemental Material, we have appended our Python script files, including a requirements list, a Pipfile, 3x Python files (.py), and 2x Python notebooks (.pynb).

### 2.5. Remote Learning Help for Students

One of the challenges in remote learning is a dependence on a students’ individual internet connectivity to the course website portal (from anywhere in the world). This course depended on success in uploading and downloading hundreds of videos up to 30 min in length, processed through the BONGO video assignment tool. Individual student issues in remote learning help were forwarded to the Computing Helpdesk.

## 3. Results and Discussion 

### 3.1. Analyses of Student Retention in a 400-Level Asynchronous, Remote Learning Oral/Written Communication and Discussion Course

Many standard courses, such as in-person laboratory courses, had been canceled for Fall 2020, ballooning pre-enrollment to 203 students for this 400-level course normally capped at an enrollment of 25 students. The final enrollment was 60 students, with 143 students de-enrolling mostly within the first few weeks of the course. Of the 203 students who had pre-enrolled and/or shadowed the course for up to many weeks, only 29.6% (or 60 out of 203) of the original number of interested, pre-enrolled students remained and completed the course and received a final grade. It was not surprising that a large fraction (70.4%) of students who sampled and shadowed the course briefly would not be agreeable to completion of the course upon discovery upon admittance that it is an intensive oral presentation and discussion course with three sets of required writing submissions in fulfillment of the course requirements.

By week six, there were a total of 62 enrolled students who submitted a written abstract and uploaded their corresponding oral presentation video on a unique, chosen research topic to the LEARN (D2L) course website. There was only a 3.2% (or two out of 62) attrition rate of enrolled students dropping out of the course after submission of their research abstract and uploading of their oral presentation video by week six. A high 96.8% retention (60 out of the 62 who completed Part A) fulfilled the second half (Part B) of the course. 

### 3.2. Analyses of Student Engagement in 400-Level Oral/Written Communication and Discussion Course Delivered Asynchronous by Remote Learning

Overall, we measured a consistent engagement in students completing required tasks throughout the 12-week course (see Table 1). In Part A of the course, enrolled students by the end of the first week of the course had all chosen a unique Systems Neuroscience research topic within the LEARN (D2L) course website out of possible research topics contained within a “*Group*”, containing a maximal enrollment of one student per each of 180 possible research topics. 

A total of 62 oral presentations were uploaded of an average length of 23.8 min out of the maximum allowable 30 min of uploaded video length after the end of Part A by Week #6. In the second half of the course, Part B, students were to post questions to the oral presenter after the peer review of oral presentations. 

While we requested a minimum of one question posed for each peer reviewed oral presentation video under the appropriate Discussion Thread for each research topic, students posed an average of 1.64 (range: 1.57 to 1.68) questions per week over the five-week oral presentation review period. Overall, there was a staggering 1985 peer review questions posted, ranging from 223 to 243 questions posed per week over the five-week oral presentation review period by the 60 or 61 students enrolled in the class.

We requested that reviewers of oral presentations fill-in peer review quiz questionnaires providing personal feedback to oral presenters and also carry out a student ranking of the four oral presentations that each student viewed per week over the five weeks. Engagement in the oral presentation review process was consistently high over the five-week oral presentation review period, with a completion rate of fill-in peer review quiz questionnaires of 95.6% (range of 92.7% to 99.6%), and a completion rate of student rankings of oral presentations reviewed per week of 87.9% (range of 77.0% to 92.3%).

There was also a consistently high rate of oral presenters filling in quiz evaluations answering whether questioners of their oral presentation had asked at least one thoughtful and unique question. This completion rate of evaluation of questioners of oral presentations was 94.8% on average, ranging from 80.2% to 94.8% per week.

### 3.3. Positive Aspects in Using the BONGO Video Assignment Tool within the LEARN Course Website for Delivery of a 400-Level Asynchronous, Remote Learning Oral/Written Communication and Discussion Course

We had to have a means for the 62 enrolled students to receive a randomized sample of 20 out of the 62 videos to peer review over five weeks in Part B of the course. Using the BONGO Video Assignment Tool, under Submit > Video Assignment > Configure >Show Advanced, individual Video Assignments could be configured to “*Automatic (System Selected)*” for Peer Review. This meant that the least viewed of the pool of 62 oral presentation videos at any point in time would automatically be sent for peer review in a student’s “*empty*” video assignment folder, that is a student’s video assignment folder lacking a video which the student had not peer reviewed yet. Once students pressed to view an oral presentation and submitted a mandatory comment in a text box to the oral presenter after viewing the oral presentation video, the next of the least viewed oral presentations would then be made available for peer review to the student in their BONGO Video Assignment folder, with a continuing of the availability of peer review of new oral presentations to peer review until the configured “*Number of Required Reviews*”, in our case 20 peer reviews of oral presentation videos, were met for every student.

A real advantage of a system that automatically populates the least watched video in BONGO Video Assignment folders is that it ensures all videos receive equal viewing and, importantly, when students drop out of the course, their oral presentation video also automatically drops out of the pool of available videos to peer review. In the end, we could give a student an oral presentation grade based on a relative ranking score of what 20 random students (~1/3 of enrolled students) felt that each oral presentation ranked in a pool of four oral presentations that each student peer reviewed every week.

### 3.4. Negative Aspects in Using the Bongo Video Assignment Tool within the Learn Course Website for Delivery of a 400-Level Asynchronous, Remote Learning Oral/Written Communication and Discussion Course

- ***Slow processing time of MP4 videos***: Each uploaded video to BONGO Video Assignment folder needed to be up to twice as long as the video’s duration for narrated PowerPoint presentation videos exported as MP4 video file to be processed by the BONGO software. We were allowing oral presentations to be submitted up to 30 min in length, which meant that uploaded videos took up to an hour to process after uploading before they could be submitted as a video assignment. A minority of students reported poor internet connectivity and were unable to process their MP4 video files through the BONGO Video Assignment Tool. **Solution**: We had told students to go to a location where they could connect to a faster more reliable internet service. We recommended that students re-process their MP4 videos to minimize video file sizes using open-sourced video creation software, such as the VideoLAN Movie Creator (VideoLAN).
- ***Compatibility issues with exported MP4 videos from PowerPoint/Keynote within PC/MAC computers***: If students used out-of-date PowerPoint/Keynote software, and older computers with out-of-date operating systems (especially MAC desktop computers/laptops), MP4 videos often would not be processed properly, and commonly the audio would be muddled or absent from the exported MP4 video of the narrated PowerPoint/Keynote file. **Solution**: A minority of students had to be forwarded to the IT Department and Computing Helpdesk to guide students through re-configuration and updates to their computers to ensure MP4 video conversion success.
- ***Videos which needed to be re-uploaded could not be repopulated to the same Video Assignment Folder*:** Students would peer review a processed MP4 oral presentation video through the BONGO Video Assignment Tool, only to realize that there were issues with it, such as the audio narration was absent or muddled. The instructor could manually “*Reset Submission*” within the BONGO Video Assignment Tool, which allowed the oral presenter to re-process and upload a newer, better version of their oral presentation video. However, once a video submission has been reset, it means the old uploaded video is removed from the pool of available videos, and any subsequent uploaded video re-enters the pool of available videos anew and is slotted in the queue to the next available student’s BONGO video assignment folder, requiring a peer review video below their peer review maximum of 20 videos. Because the videos are populated automatically in student Video Assignment Folders by BONGO, we were not able to re-assign the newer, updated oral presentation video to the student who had already been assigned and previously peer reviewed the older, previous version of the oral presentation video. It meant that this peer reviewer would have to evaluate an additional, random oral presentation video *in lieu* of the one that they had peer reviewed but was no longer in their available pool of videos to peer review.
- ***Concerns that students would pre-select the order in which they peer reviewed their allotment of 20 oral presentations*.** Students were to rank four oral presentations per week over five weeks, but BONGO Video Assignment Tool only permitted the setting of the number of required oral presentation videos that each student had to peer review in total, such as viewing of 20 videos, but we could not set limits on the access to oral presentation videos, which were fed to student BONGO Video Assignment folders on a weekly basis. This meant that students had access to view their quota of 20 peer review oral presentations all at once, and we could not configure BONGO Video Assignment Tool to restrict student peer review to a maximum viewing of four oral presentations per week. To prevent students from viewing all 20 videos and then choosing the four oral presentations out of the 20 in which to evaluate, we needed to increase the anonymity of the oral presentations so that students could not choose and select the order in which they viewed their oral presentations. **Solution**: We could choose “*Conceal reviewer identity*” and “*Conceal submitter identity*” under the *Show Advanced* configuration options to ensure that the identity of the submitter and the peer reviewer of the oral presentation video populated in every student’s BONGO Video Assignment Folder were anonymous. Since the icon of each oral presentation video was identified by a photo of the first slide, we required students to mask their video with an instructor-provided common, general first slide, with a subsequent, second slide template provided which contained identifying details such as the author of the oral presentation, an author-selected oral presentation title, and the instructor-provided unique research topic number and the associated title of the chosen research topic provided under the research topic group. This meant that students did not know of the identity of the oral presentations that they were going to view until after they had mouse-clicked on the oral presentation icon and started the oral presentation to view.
- ***Peer review comments left under the BONGO Video Assignment Tool under the “Automatic (System Selected)” peer review method are limited in visibility to the oral presenter, the reviewer, and the instructor*:** There needed to be peer reviews from 20 students for each oral presentation video, and each peer reviewer was required to submit *at least one thoughtful and unique question* to each oral presenter. Because of a configuration that limited private viewership of peer review comments to the oral presenter and instructor only within the BONGO Video Assignment Tool, we could not use the BONGO Video Assignment Tool for students to post their *thoughtful and unique question(s)*, because as many as 19 different peer reviewers needed to be able to view the previous questions posed by students during previous peer reviews of an oral presentation. **Solution**: We had students post their *thoughtful and unique question(s)* outside the BONGO Video Assignment Tool, using the “Reply” function under each unique Discussion Thread containing the associated research abstract and appended PDF formatted files of relevant scientific papers for each research topic within the LEARN (D2L) course website. While our solution required students to jump between the BONGO Video Assignment Tool to watch their videos and to a Discussion Thread within the LEARN (D2L) course website to pose research questions in response to watching and analyzing the BONGO video, students quickly became accustomed to switching back and forth between the Discussion Threads within the LEARN (D2L) course website and the available videos provided within folders generated by the BONGO Video Assignment Tool. The Discussion Thread of every research topic became the hub of activity where research abstracts and PDF download of scientific papers were located. The students’ criticism towards this approach was that there were at least 20 questions posed by students under the Discussion Thread assigned to each research topic by week five of peer reviews, creating a long thread of responses, which students found irritating to scroll through.
- ***Formatting of the Video Assignment folder containing the oral presentations to “Automatic (System Selected)” meant that the oral presentation videos were private to select peer reviewers, and we needed to create an additional five open access “Use Manual (Learner Selected)” BONGO Video Assignment Folders, where students could populate video responses to peer review questions posed by the four students assigned to peer review their oral presentation video every week.*** The problem in having six different BONGO Video Assignment folders required for uploading of videos totaling many hundreds of videos for the class was that at least one or more students mistakenly populated their video in the wrong folder. BONGO lacks an overview function for the instructor to identify where a particular student’s video has been misplaced amongst the six different BONGO Video Assignment folders. Videos for every student are sorted under each Video Assignment Folder, but a general indexed search cannot be made by a student to identity which Video Assignment Folder a particular student had mistakenly uploaded a video to.
- ***Because peer reviews of oral presentations were populated randomly and privately to the inbox of select peer reviewers, this meant that each student had access to only 1/3 (20 out of ~60 students) of submitted oral presentations of the class.*** We should have had students upload an identical copy of their oral presentation video to a separate BONGO Video Assignment folder that was peer review formatted as open access: “*Manual (Learner Selected)*”, in addition to a separate Video Assignment folder that was configured to “*Automatic (System Selected)*” and limited in viewership to those required to review the oral presentation video. The open access: “*Manual (Learner Selected)*” formatted BONGO Video Assignment folder would have provided a repository for interested students to view oral presentation videos for everyone in the class, outside the 20 oral presentations that each student was required to peer review.
- **A lack of integration of the BONGO Video Assignment Tool with instructor tools within the LEARN (D2L) course website meant that it was difficult to track ongoing student engagement and participation in the course**. BONGO Video Assignment Tool randomly populated each student with 20 oral presentations to evaluate. Because of incompatibility of the BONGO Video Assignment Tool and instructor tools within the LEARN (D2L) course website, it was not easy to track whether students who had posed a question under a Discussion Thread within the LEARN (D2L) website corresponded to the appropriate oral presentation video which they were required to view within a BONGO Video Assignment Folder. Moreover, it was not easy to track a student’s video response submitted to a BONGO Video Assignment Folder addressing questions corresponding to questioners posed under a student’s Discussion Thread within the LEARN (D2L) website. For example, students would query to the instructor or two teaching assistants why they had more or less than the four students required to pose questions every week under their Discussion Thread within the LEARN (D2L) website. We were not easily able to retrieve this information for students. Data is not easily mined without cross-compatible, indexing functions between the 1308 posted questions in the Discussion Thread category within LEARN (D2L), with 2400 peer evaluations stored in the category of different online quiz folders in LEARN (D2L) and with the 363 video uploads populated in six different BONGO Video Assignment Folders.
- **The appropriate location for students to upload their BONGO Video Assignments was counterintuitive.** Students could not upload their video assignments in the LEARN (D2L) course site of what appeared to be labeled as the correct location to upload their video: > Submit > Video Assignment. “*Submit Video Assignment*” provided students with an overview of the titles of video assignments which were due, the type of project that was due, and a potential due date for the submission of video assignments. The real location to submit video assignments was an instructor created folder under a “Contents” subfolder on the LEARN (D2L) course website. Even though instructions for where to upload video assignments within LEARN (D2L) course website were made available in an uploaded HOW-TO video and in the contents of reminder emails to all students in the course, there were many students who contacted the instructor describing what they assumed was a unique problem in not being able to submit their assignments by means of the pull-down menu on the LEARN (D2L) course website, appropriately labeled “*Submit Video Assignment*”.

### 3.5. Overall Course Feedback from Students on Remote, Asynchronous Delivery of a 400-Level Oral Discussion/Presentation and Written Communication Course

#### 3.5.1. Students Appeared to Prefer the Asynchronous, Remote Format for the Oral/Written Communication Course and Found the Course to Be of High Educational Value but Also a Lot of Work to Complete

Surprisingly, a majority of students who answered the course evaluation survey (see Table 2 and Appendix M—Sample student feedback comments for the course) preferred delivery of this oral and written communication course by a remote, asynchronous format, and would recommend for future students to take the course in an asynchronous, remote format in the same manner that they did, rather than to enroll in the same course offered as a standard “*Live*” in-class, oral presentation, and group discussion course.

The students’ preference for a remote, asynchronous, delivery of this communication course appears to largely relate to the comfortability of students in being able to deliver a narrated oral presentation without the anxiety in having to perform in front of a live audience. Moreover, the asynchronous delivery format gave students many days to upload a video response to questions posed of their oral presentation. The asynchronous delivery format provided students with time in which to research and prepare thoughtful answers to questions posed to them, without feeling intimidated to respond to questions unprepared and under the stress of a live audience.

What these students were not able to judge in their assessment of the remote, asynchronous learning format is the absence of instructor input in the remote learning format. A professor is more able to facilitate, monitor, and guide student learning and discussion within the in-class “live” version of the course. Some students left course feedback wishing that they had received more guidance in how to research their chosen topic, and in communicating scientific research, both orally and written. While many students expressed in anonymous feedback of the course how much they appreciated the self-learning opportunities in the course, and the peer engagement in a wide variety of research topics, other students found that this more self-learning approach does not suit their learning style.

Most students who answered the course feedback survey felt that the course was in the category of “more” or “most” difficult of the courses that they were taking at the same time, with almost all believing the course entailed a “heavy” to “heaviest” workload compared to the other courses they were taking. While complaining about the heavy workload of the course, a student did remark that they did not “*realize just how much* [that they had] *learned”* until the course was over. 

Students who opted to submit course feedback were nearly unanimous in their statements about the “high” or “very high” educational value of the course, and felt that they were more interested in carrying out research in a Master’s or PhD graduate degree after having carried out an individual research project investigation and having contributed to research discussions in this course.

#### 3.5.2. Students’ Approval of Online Learning and the Asynchronous Delivery of a Communication Course Is Consistent with Outcomes Reported by Many Studies

There are reported benefits and drawbacks of online learning [10,11], and the degree of success appears to relate to how well the technology is employed to enhance engagement and interactivity, over in-person learning opportunities for enrolled students [12,13]. Having students carry out preparatory learning on their own through research, reading, and watching videos, and then having them engage later to discuss what they have learned in a group classroom setting, whether in-class or virtually, creates the so-called “flipped classroom” that can result in significant improvement in student learning compared with traditional teaching methods [14,15,16]. 

The use of asynchronous videos for the virtual classroom appears to work best when: (a) there are strict guidelines and deadlines that drive student interactivity, (b) when the instructor facilitates but does not dominate the classroom discussion, and (c) the students receive regular feedback on their class performance during the school term [17,18,19,20,21]. A general consensus is that course content created by student-produced video assignments generates more student engagement and learning opportunities than more passive learning in watching and learning from instructor created videos [22,23,24]. 

#### 3.5.3. Some Students Encountered Serious Problems Converting Their Narrated PowerPoint Presentations into Exported MP4 Videos, and Processing Uploaded MP4 Formatted Videos within the BONGO Video Assignment Tool

Outside complaints of the self-learning approach not suiting every student’s learning style and the workload burden of the course, another category of major complaint towards the course involved formatting issues of MP4 files and time for uploading and conversion of videos using the BONGO Video Assignment tool. One quarter of students who answered the course feedback survey reported having serious internet connection issues and/or MP4 formatting issues that hampered their ability to submit BONGO Video Assignments on time.

#### 3.5.4. Students Were Understandably Fearful of a Peer Review Ranking System of Their Oral Presentations

Many students expressed a hesitancy in that 35% of their final grade was being assigned through a peer ranking system of their oral presentations, which did not involve instructor authority over assigning of grades. Students take the peer ranking system seriously and were highly consistent in their choice involving which of the oral presentations they ranked highly and those which they assigned low ranking scores. 

For example, one of the highest ranked oral presentations was ranked “*Best*” (11 times), “*Second Best*” (five times), “*Third Best*” (two times), and “*Least favorite*” (one time). This is a student who was consistently ranked “*Best*” or “*Second Best*” oral presentation by 84.2% (that is 16 of 19) of different peer reviewers of the four oral presentations which students viewed in a week.

One of the lowest ranked oral presentation was ranked “*Best*” (zero times), “*Second Best*” (one time), “*Third Best*” (six times) and “*Least favorite”* (11 times). This is a student who was ranked in the bottom two categories “*Third Best*” or “*Least favorite*” oral presentation by 100% (that is by 17 of 17) of different peer reviewers of the four oral presentations which students viewed in a week.

Because of minor differences in the number of reviewers received for each oral presentation, ranging from 16 to 20 reviewers (average = 17.6), ranking scores were tabulated as a summation of scores in each of the four categories (“*Best*” “*Second Best*” “*Third Best*”, and “*Least favorite*”) normalized as a fraction over the total number of reviews which each student received for their oral presentation.

#### 3.5.5. Despite the Fearfulness of Students in Use of a Peer Review Ranking System, There Was a Strong Correlation Between the Student Peer-Graded Work and the Independently-Graded Instructor-Graded Work

**Figure 1:**
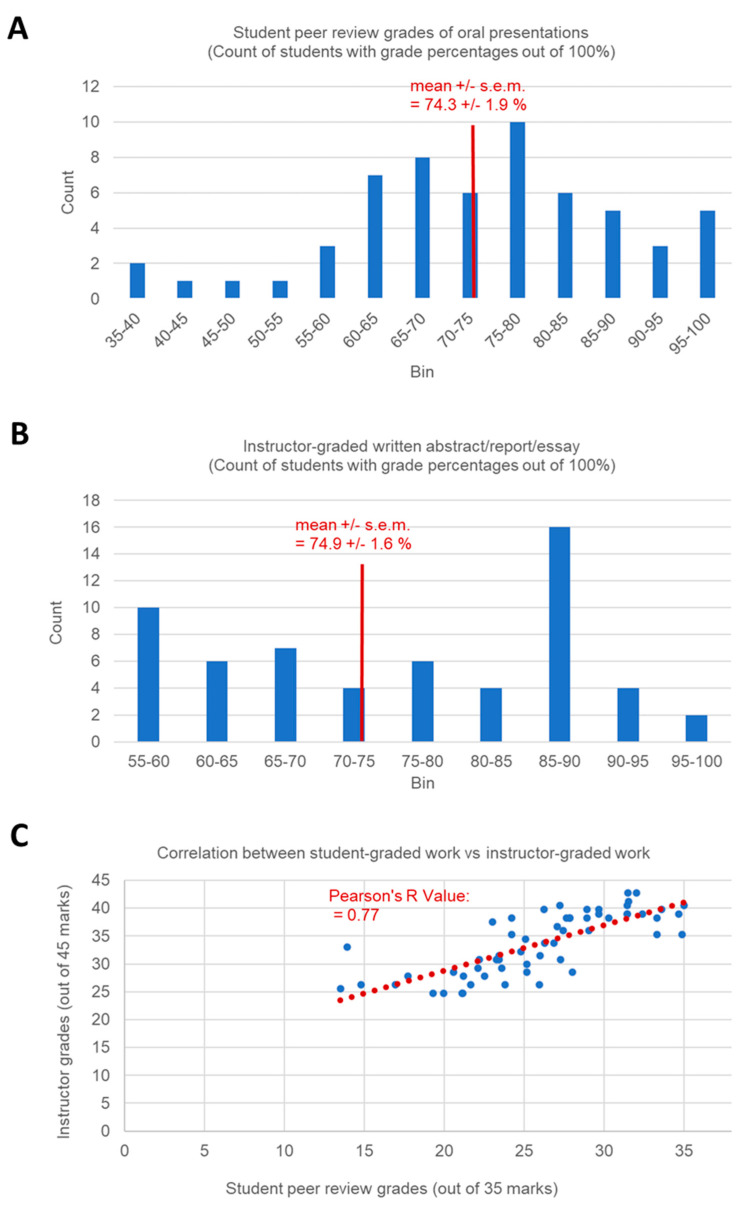
Strong correlation between student-graded and professor graded work. (**A**) Frequency histogram of marks (out of 100%) graded by student peer review; (**B**) Frequency histogram of marks (out of 100%) graded by instructor; (**C**) Plot of the correlation between the instructor graded work (out of 45 marks) versus the student-graded work (out of 35 marks).

The student peer review rankings of oral presentations (worth 35% of the final grade) were scaled to a mean student score of 74.25 +/− 1.96%, with a minimum score of 38.6% to a maximum score of 100% for n = 60 enrolled students (Figure 1a). The instructors and teaching assistants did not have time to view and evaluate each of the videos uploaded by students, given the total volume of uploaded videos submitted by students. There were >363 total video uploads by students consisting of oral presentation videos (63 narrated Powerpoint presentation videos of up to 30 min in length each, totaling up to a maximum of 30 h of oral presentations), as well as >300 videos submitted of responses to student questions of each peer viewed oral presentation (of different video lengths).

The independently graded written work marked by the instructor (worth 35% of the final grade) consisting of a submitted research abstracts, research reports on students’ chosen research topics, and essays on the research topics provided to them (each piece of written work was 15% of the final grade). The frequency histogram illustrating the spread of grades reveals a less than normal distribution of instructor marked grades (Figure 1b), compared to the student peer reviewed grades (Figure 1a), with a tendency of scores clumped into a very high bin (85–90%) resulting from many students’ strong written work submitted in the excellent category (16 students) and a clumping of grades in the lowest possible bin (55–60%) (10 students), in the barely acceptable range of what was poorly written submitted work. While the instructor-graded work did not follow a typical normal distribution of grades, the mean +/− s.e.m. = 74.9 +/− 1.6% of instructor-graded work (Figure 1b) was nearly identical to the student peer graded work (mean +/− s.e.m. = 74.3 +/− 1.9%). The calculated Pearson’s Correlation Coefficient (R value) indicates a strong correlation of 0.77, between the student peer review graded work and the instructor-graded work (Figure 1c), despite the fact that the instructor marked the written work without the instructor having examined the submitted narrated oral presentations, or submitted video responses to peer questions, or knowing the peer ranking of student oral presentations when marking the written work. This suggests that the students who tended to be ranked favorably by student peer review were also the students who tended to submit the strongest written work for the course. The strong correlation between the student peer review graded work and the instructor-graded work indicates that the students were able to objectively evaluate oral presentations for their scholastic merit, closely corresponding to how the instructor independently assessed the depth and strength of the student’s research, as revealed in the submitted written work.

## 4. Conclusions

While a remote, asynchronous learning approach is non-ideal, and will never replace an instructor who is more able to directly facilitate learning and discussion in a “*live*” classroom with small enrolments (<15 to 25 students), there were (to our surprise!), real advantages in delivering an oral/written communication course remotely and asynchronously. 

(1) Student accessibility is the greatest advantage of a remote, asynchronous learning delivery of an oral/written communication course. Normally much of in-class time is devoted to the “live” delivery and discussion of each student’s oral presentations, one-by-one, to a maximum of 25 students, staggered over the last six-weeks of the course. In the remote, asynchronous delivery format, we were able to have all of the ~60 enrolled students bank their narrated oral presentations in video format by the same deadline date at the end of the first half of the course by Week #6. Then in the last half of the course (Part B), we were able to set instructions to enforce engagement and peer evaluation of enrolled students into a total of 1200 unique *virtual classrooms* consisting of an oral presenter and peer reviewers of oral presentations every week, over a time span of five weeks. 

(2) Asynchronous and remote delivery of the course meant that students could deliver their oral presentation and respond to peer questions of their oral presentation at their leisure and convenience, without undue stress and anxiety associated with delivery of a “live” performance in front of their peers. 

(3) In a standard in-person class, we would have time set aside over a few minutes for a few students to field questions to an oral presenter (*in what was usually an awkward exchange between students because of the typical nervousness of students*) in a “live”, oral performance of student presentations in class. In the remote, asynchronous format, each student could receive *unique and thoughtful* questions from 20 different students who viewed their narrated oral presentation video. Additionally, since it was a process where students responded to these 20 *unique, thoughtful* questions posed by students stretched over a period of five weeks, it provided opportunities for each student to steadily increase the depth of understanding of their own research topic through sequential peer engagement over a five-week period after they needed to research, prepare, and upload their narrated oral presentation onto the course website.

(4) A remote, asynchronously delivered course can facilitate much more peer communication than in a “live” oral presentation/discussion course. There was a total of 62 oral presentations and 301 uploads of video responses to 1985 questions posed by peers over the 12-week course. There was a total of 1159 feedback questionnaires and 1066 student rankings submitted for peer review of oral presentations, and 1091 evaluations submitted by oral presenters evaluating the quality of questions posed by reviewers of oral presentations. 

(5) A drawback in the remote, asynchronous delivery of the course is that most of the peer engagement is blind to the single instructor and two teaching assistants, based, in large part, on the enormity of the peer engagement through the internet and course website portal, consisting of hundreds of hours of uploaded student videos and thousands of questions posed by enrolled students and thousands of submitted peer evaluations. Compounding the issue is the inability of instructors or the two teaching assistants to cross-index data linked between the student entries in the LEARN course website and the uploaded videos stored within the BONGO Video Assignment tool. The weighting of peer evaluation of oral presentations and quality of questions posed of oral presenters was limited to 35% of the total grade for the course, since the instructor and teaching assistants were mostly blind to the depth and quality of the peer engagement and evaluation of oral presentations. 

The consistent engagement of students, and the positive feedback from enrolled students in the course, despite its remote, asynchronous delivery, indicate that a future version of this oral/written discussion course continue to be delivered remotely and asynchronously, in whole or in part, even without a mandated delivery of the course by a remote and asynchronous method due to the COVID-19 pandemic restrictions in 2020–2021.

## Figures and Tables

**Table 1:** Assessment of student engagement in 400 level oral presentation and discussion course carried out asynchronously by remote learning.

	Part A	PART B (Peer Review Period of Oral Presentations)					
	Week #6	Week #7	Week #8	Week #9	Week #10	Week #11	Total
**Task Carried out by Students**	**Oral Presentation Submissions**	**Video Responses Uploaded to Peer Review Questions of Oral Presentations**					
# of video uploads/expected # of video uploads (%)	62/62 (100%)	61/61 (100%)	61/61 (100%)	61/61 (100%)	60/60 (100%)	58/60 (96.7%)	363/365 (99.5%)
**task carried out by students**		**Questions posed by peer reviewer of oral presentations**					
average number of questions posed by peer review of oral presentations (expectations of minimum of one)	N/A	1.57	1.68	1.67	1.67	1.60	1.64
total number of questions posed by peer review of oral presentations (expectations of minimum of one)	N/A	383	410	407	400	385	1985
**task carried out by students**		**Peer review feedback questionnaires completed for oral presentations**					
average/expected average of feedback questionnaires received for each oral presentation	N/A	3.98/4.00 (99.6%)	3.94/4.00 (98.4%)	3.68/4.00 (91.9%)	3.82/4.00 (95.6%)	3.71/4.00 (92.7%)	3.83/4.00 (95.6%)
total number of feedback questionnaires received for each oral presentation	N/A	243	240	224	229	223	1159
**task carried out by students**		**Peer review student rankings completed for oral presentations**					
average/expected average of student rankings received for each oral presentation	N/A	3.69/4.00 (92.3%)	3.61/4.00 (90.3%)	3.65/4.00 (91.1%)	3.55/4.00 (88.7%)	3.08/4.00 (77.0%)	3.52/4.00 (87.9%)
total number of student rankings received for each oral presentation	N/A	225	220	222	213	185	1066
**task carried out by students**		**Peer review student evaluations of whether oral presenters believed that questioners posed a *thoughtful, unique* question of oral presentations** **that they reviewed**					
average/expected average of peer review evaluations of questioners of oral presenters	N/A	3.76/4.00 (94.0%)	3.79/4.00 (94.8%)	3.73/4.00 (93.1%)	3.52/4.00 (87.9%)	3.21/4.00 (80.2%)	3.60/4.00 (94.8%)
total number of peer review evaluations of questioners of oral presenters	N/A	229	231	227	211	193	1091
total number of tasks carried out by students	62	1142	1162	1142	1113	1043	5664

**Table 2:** Course evaluation feedback from enrolled students on 400-level, remote, asynchronous delivery of an oral discussion/presentation and written communication course (n = 16).

#	Question	Student Responses (%)
1.	Would you recommend the remote and asynchronously delivered version of this 400-level oral discussion/presentation and written communication course for other students to take?	
	**I recommend the remotely and asynchronously version of this course for future students to take.**	**69.8%**
	I DO NOT recommend the remotely and asynchronously version of this course for future students to take.	18.8%
	I AM NOT SURE if I would recommend this version of the course that I took	12.5%
2.	Would you have preferred to have taken this course as a “live” in-class Group Discussion course or do you prefer the remote participation approach?	
	I would prefer to have taken this course as a “live” in-class Group Discussion course	31.3%
	**I prefer the remote participation, asynchronous learning approach for this course.**	**50.0%**
	I AM NOT SURE if I would recommend this version of BIOL 476 that I took	18.8%
3.	Did you have serious internet connection issues and/or MP4 formatting issues that hampered your ability to submit BONGO VIDEO ASSIGNMENTS on time?	
	YES, I HAD serious internet connection issues and/or MP4 formatting issues.	25.0%
	**NO, I DID NOT HAVE serious internet connection issues and/or MP4 formatting issues.**	**75.0%**
4.	Did this 400-level oral discussion/presentation and written communication course require more, less or the same amount of work than other courses that you have taken?	
	**This 400-level course required MORE WORK than most other courses that I have taken**	**81.25%**
	This 400-level course required LESS WORK than most other courses that I have taken.	6.25%
	This 400-level course required about the SAME AMOUNT OF WORK than most other courses that I have taken.	12.50%
5.	Are you more likely to consider continuing in research in a Masters or PhD graduate degree from having carried out a project involving research topic investigations and group discussions in this course?	
	**I am MORE likely to consider continuing in research in a post-graduate degree having taken this course**	**68.75%**
	I am NO MORE likely to consider continuing in research in a post-graduate degree having taken this course	31.25%

The color and bold highlight the response most chosen by students in the questionnaire.

**Figure A1:**
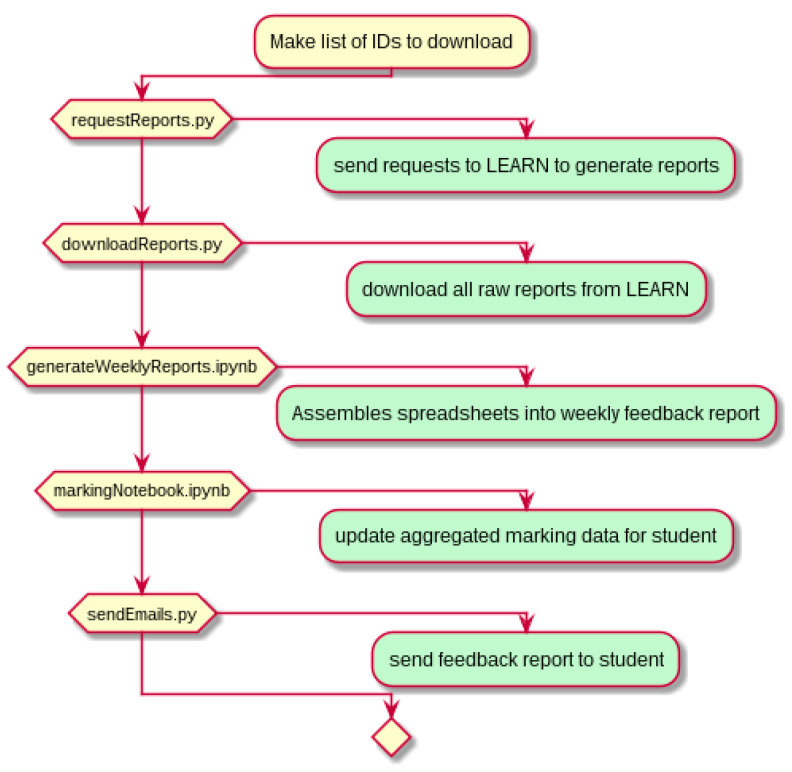
Flow chart of Python script sequences.

**Figure A2:**
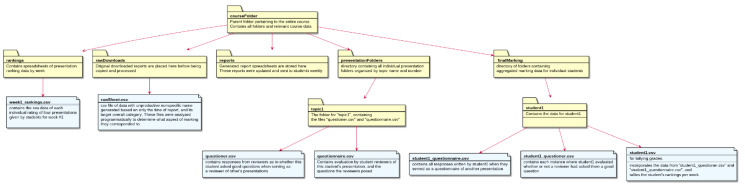
Flow chart of folder structure.

## Data Availability

All data presented in this study are available within the research article and supplementary materials.

## Supplementary Materials

The following are available online at https://www.mdpi.com/article/10.3390/brainsci11060693/s1, Python associated script files for automatically sending weekly peer review feedback reports to enrolled students:

requirementsList

Pipfile

clean_downloadReports.py

clean_generateWeeklyReports.ipynb

clean_markingNotebook.ipynb

clean_requestReports.py

clean_sentEmails.py

## Appendix A. Listing of Available Research Topics and Instructions in How to Choose a Research Topic

How to choose a research topic:

- Under Connect > Groups > View Available Groups > Join Group. Each Group is a research topic, with a maximum enrollment of one student.

**Table A1:** Available research topics (001 to 0180).

001	Adolescent brain and the developing prefrontal cortex
002	Altered chromatin synaptic function, neuron-glial signaling and the genetics of autism spectrum disorder
003	Aplysia and synaptic plasticity
004	Astrocyte function and drug access to the blood: brain barrier
005	Axonal growth and axon guidance
006	Biological clock mechanisms of Drosophila and mammals
007	Central pattern generators and locomotion in cats
008	Circuitry of addiction and reward
009	Classification of neurons and synapses using genetic and molecular markers
010	Connectomics
011	Critical periods in visual development
012	Dendritic branching, spine dynamics, and dendritic stability
013	Dopamine, alpha-synuclein, basal ganglia, and Parkinson’s disease
014	Epigenetics and sex differences in the brain
015	Evolution of synapses, nervous systems, and the cerebral cortex
016	FOXP2 and vocal communication
017	GABAergic synaptic inhibition and excitation
018	Gal4/UAS and other genetics tools used in zebrafish and fruit fly models
019	Gamma oscillations and brain synchrony/asynchronous rhythms
020	Glia and astrocyte signaling
021	Glutamatergic silent synapses during brain development, addiction and synaptic plasticity
022	Gut microbiome, germ free mice and its influences on brain and behavior
023	Hearing, hair cells and tinnitus
024	Hypothalamic feeding circuits, satiety and body weight
025	Mauthner Cells, the vestibular system, and tail-flip escape behavior
026	Mechanisms of action of endo-cannabinoids
027	Mechanisms of epileptogenesis and treatment
028	Mirror neurons
029	Molecular mechanisms and treatments for Alzheimer’s disease
030	Multiple sclerosis: inflammation, autoimmunity, and degeneration
031	NMDA receptor subunit diversity, synaptic plasticity, and disease
032	Nanodomain coupling in fast mammalian synapses
033	Navigation by rats versus bats
034	Nerve repair and regeneration
035	Nervous system dysregulation and schizophrenia
036	Neural adaptations in birdsong
037	Neural circuits underlying thirst and fluid homeostasis
038	Neural mechanisms of gustation and taste recognition memory
039	Neurexins-neuroligins in synaptic identity, synapse formation and autism
040	Olfactory signaling and pheromones
041	Optogenetics
042	Oxytocin/vasopressin, knockout animals and the social brain
043	Phantom limb pain and sensorimotor function
044	Plasticity and stability of visual field maps in the primary visual cortex
045	Polyglutamine spinocerebellar ataxias—from genes to potential treatments
046	Prion proteins and memory
047	Saccadic eye movements and visual perception
048	Sodium and calcium channels and pain
049	Specificity of synapse formation
050	Speech execution, perception and processing
051	Spike timing dependent plasticity (STDP) and brain metaplasticity
052	Synaptic maturity and assembly
053	Synaptic plasticity and the hippocampus
054	Synaptic tagging and capture in memory formation
055	The Leech sensory-motor network
056	The aging brain and sirtuins
057	The asymmetrical brain (lateralization
058	The neuron doctrine, variability, compensation, and homeostasis in neuron and network function
059	Topographical mapping in the visual system
060	Transcriptional regulation of photoreceptor development and homeostasis in the retina
061	Computational approaches in understanding visual body perception and facial identity
062	WNT signaling in developmental patterning to neuronal connectivity of the nervous system
063	Whisker-Barrel cortex
064	Color vision processing
065	ribbon synapse diversity in sensory neurotransmission
066	Emerging connections between cerebellar development, behaviour and complex brain disorders
067	Autophagy in acute brain injury
068	The use of brain organoids to investigate neural development and disease
069	The dynamic nano-architecture of the axonal cytoskeleton
070	Mechanisms of sound localization
071	SHANK proteins: roles at the synapse and in autism spectrum disorder
072	Mitochondria at the neuronal pre-synapse in health and disease
073	Genetic and activity-dependent mechanisms underlying interneuron diversity
074	Backpropagating action potentials and synaptic plasticity
075	Stem cell therapies for Parkinson’s disease
076	Roles of axon guidance molecules in neuronal wiring in the developing spinal cord
077	Comparison of the retinal basis of vision across the animal kingdom
078	How the epigenome reshapes the synapse
079	The neural and computational systems of social learning
080	Immune cell regulation of glia during CNS injury and disease
081	Oscillating circuitries in the sleeping brain
082	Nanophysiology: regulation of ionic flow in neuronal subcompartments
083	Connexins and pannexins in health and disease
084	Calmodulin kinases and autophosphorylation in learning and memory
085	Role of synaptotagmins pre-synaptically and post-synaptically
086	Programmed axon degeneration: from mouse to mechanism to medicine
087	Mechanisms underlying gain modulation in the cortex
088	Striatal circuits for reward learning and decision-making
089	Dentate gyrus circuits for encoding, retrieval, and discrimination of episodic memories
090	Diverse forms of GABAergic synaptic plasticity
091	Dendritic structural plasticity and neuropsychiatric disease
092	Macroscopic gradients of synaptic excitation and inhibition in the neocortex
093	Synaptic pruning mechanism in neural development
094	Backprop error correction learning in biological nerve networks
095	Role of Two-Pore K2P channels in health and disease
096	Acid-sensing ion channels (ASIC) in neural function and disease
097	Insulin-Like Growth Factors (IGFs) in the developing brain
098	B cells in neurodegenerative brain disorders
099	Stress granules, RNA-binding proteins, and the pathophysiology of neurodegenerative diseases
100	Optogenetic odors and coding logic of olfactory perceptions
101	Transient receptor potential cation channels (TRP) in degenerative disorders of the PNS
102	Neural mechanisms regulating torpor and hibernation
103	Homeostatic plasticity in tuning a neuron’s action potential duration
104	Tuning of a mother’s temporal association cortex by pup’s ultrasonic vocalizations
105	Transplanting neural progenitor cells for treatment of spinal cord injury
106	Targeting aquaporins in treating central nervous system edema
107	Visual cortical prosthesis (VCPs) in treatment of acquired blindness
108	Computational models of dendritic function
109	The brain-spleen link
110	Visualization of synaptic memory codes in the brain
111	The origins of parenchymal and non-parenchymal macrophages in the brain
112	Encoder-decoder systems for human speech
113	Calcium activated potassium channels in health and brain disease
114	Subcortical sensory processing
115	Lateral habenula as the brain’s antireward center
116	The dorsomedial hypothalamus (DMH) and psychosocial stress
117	Skin temperature-sensing mechanisms
118	Cross-modal object recognition between visual and tactile senses
119	Oscillatory circuits in the cortex during reward guided behavior
120	The ventromedial hypothalamus in the regulation of aggression
121	Ventromedial hypothalamus and aggression
122	Neural circuits underlying mating and egg laying in oviparous females
123	Controls of neurovascular coupling (NVC
124	Neurogenesis in the olfactory neuroepithelium
125	Monosomes and the local translation of mRNAs in the neuropil
126	Kinesins in learning and memory
127	Paraventricular thalamus in arousal and valence processing
128	Temporal patterned input influences on motor control
129	Fear extinction memories in the basolateral amygdala
130	Oligodendrogenesis and memory
131	Diversity and functions of retinal ganglion cells
132	Neural control of food intake behavior
133	Huntington disease (HD) and huntingtin
134	Rho-family GTPases in the coordination of axonal growth
135	Spatiotemporal patterns of neural activity and expression of inducible transcription factors (ITFs)
136	Alterations in synaptic vesicle quantal quantity and size in brain-related disease
137	Uncovering of neuronal and glial cell diversity using single cell RNA sequencing and split-pool barcoding
138	Synaptic interactions between neurons and tumors which regulate tumor growth
139	Astrocytes and chronic itch
140	Melanin-concentrating hormone-expressing neurons (MCH) and the regulation of sleep, wakefulness, and memory
141	Transcriptional network in the pre-frontal cortex which drives stress resilience
142	Use of iPSC and iCRISPR technologies in the investigation of human brain diseases
143	Reprogramming of reactive glial cells into cortical neurons
144	Super-resolution imaging approaches in neuroscience
145	Neuropeptide regulation of motivations
146	Dorsal raphe nucleus regulation of thermoregulation
147	Mechanisms of chemotherapy-related cognitive impairment (CRCI)
148	Perineuronal nets (PNN) and implications for memory and psychiatric disorders
149	Object-vector coding in the medial entorhinal cortex
150	Heterogeneity in serotonin (5-HT) signalling and targeted therapies for serotonin-related disorders
151	The role of NEUROD1 in neuronal reprogramming
152	Calcium influx driving axon degeneration in multiple sclerosis models
153	Somatosensory feature-selective encoding of vibrations
154	Mechanosensitive channels and neuronal outgrowth after nerve injury
155	The role of K+/Cl- co-transporters in brain development and recovery from nerve injury
156	Neural mechanisms underlying brown adipose tissue thermogenesis
157	Role of mitochondria in regulation of neural stem cell (NSC) fate
158	Control of release probability at nerve terminals
159	Peripheral clock regulation of the master clock within the suprachiasmatic nucleus (SCN)
160	Genetic basis of cortical folding
161	Orexin neurons and narcolepsy
162	Nonsense-mediated RNA decay (NMD and influences on neuronal development, axon guidance & synaptic plasticity
163	The use of genetically encoded calcium indicators (GECIs) to measure cortical layer specific axon activity
164	Sharp wave-ripples (SWR), memory retrieval, and memory consolidation
165	Homeostatic control of spontaneous activity in the developing auditory system
166	Maintenance, reserve, and compensation in the aging brain
167	The resting membrane potential in driving neuronal phenotypes during brain development
168	Maternal inflammation during pregnancy and in utero brain development
169	Medial temporal lobe and involvement in brain encoding of symbolic numbers or numerosity
170	Brain parcellation approaches to define spatial heterogeneity in the brain
171	The ubiquitin-proteasomal system and amyotrophic lateral sclerosis (ALS)
172	The role of the 3’untranslated region (3’UTR) of genes in synaptic stability and plasticity
173	Synaptic (homeostatic) scaling
174	Sleep need index phosphoproteins (SNIPPs) and sleep deprivation
175	Lifetime cortical myelin plasticity and age-related degeneration
176	The preBotzinger Complex (preBotC) regulation of breathing
177	Notch signaling and cortical neurogenesis
178	Electrocommunication and electrolocation in electric fish
179	Neurological and neuropsychiatric complications of COVID-19
180	Role of neuropeptide Y in neurogenesis

## Appendix B. Instructions in How to Research Your Research Topic (Over Weeks and Weeks of Preparation)

Start by reading and making notes from general review articles on your research topic:

- Go to high impact review journals: “Nature Neuroscience Reviews” or “Current Opinion in Neurobiology” or “Annual Reviews in Neurosciences” to find your topic, or go to PUBMED:
- https://pubmed.ncbi.nlm.nih.gov/ (accessed on 24 May 2021) and restrict your search to “Review”
- After reading many review articles, you will gain a broad perspective of what encompasses the research topic. You will get a sense of what are the burning scientific questions (i.e., hypotheses) that are pursued, and differing opinions of researcher groups. Take notes of references of experiments from original scientific research manuscripts at the back of the review articles that provide key evidence for developing the key opinions/hypotheses. You will be questioned by other students in the class, and it is better to have a wide base of knowledge around your topic in order to be able to provide intelligent responses to good questions from other students.

Read and make notes from original scientific research manuscripts on your research topic:

- Read selected original scientific research manuscripts from beginning to end (Abstract, Introduction, Experimental Procedures/Materials and Methods, Results, Discussion, Conclusion).
- Contained in the results section of original manuscripts are graphs and tables that provide key results that shape the opinion on the research topic. Make note of the particular figures/tables of these original manuscripts. You have to explain at least three experimental figures from different research manuscripts within your oral presentation.

You must choose research manuscripts which are at least impact factor 4.0 and above—preferably choose research manuscripts above impact factor 8.

- The most widely read and impactful research is in journals such as: Cell, Nature, Science, Proceedings of the National Academy of Sciences, USA.

## Appendix C. Instructions in How to Write your Research Abstract

(1) ABSTRACT SUBMISSION: Must not exceed 750 words.
  - Preparation and submission of an abstract in Nature Journal format
  - Link to: https://www.nature.com/documents/nature-summary-paragraph.pdf (accessed on 24 May 2021)
(2) ORGANIZATION OF THE ABSTRACT:
  - GENERAL: You want to start by providing a broad introduction to the neuroscience topic (stating in one to a few sentences)
  - GENERAL->SPECIFIC: Then state the scientific questions of the field addressed by particular scientific studies
  - SPECIFIC: Then summarize the key experimental findings that address the particular questions
  - SPECIFIC->GENERAL: Put these experimental findings into a more general context of how these findings shape the current opinion within the neuroscience topic
  - GENERAL: Then put these results and other results like it into a broader perspective, the future outlook of the research topic.
(3) SUBMIT REVIEW MANUSCRIPT: (submit one, maximum two) key review article(s), that best encompasses the general opinion/perspective of the research topic (encompassing the topic theme described in your abstract)
(4) SUBMIT ORIGINAL SCIENTIFIC REARCH ARTICLES: provide manuscripts for 3 to 5 original research articles, illustrating and explaining the methods and key experiments on key figures/graphs within the research articles that address the opinion/perspective of your research topic.

Week 5, one week before the final deadline submission date for your abstract, will be a deadline to submit a draft of your research abstract.

Post your abstract and spend a week editing and sharing your research abstract for others to peer review your submitted abstract for you in preparation for submitting the final copy of your abstract by week 6.

## Appendix D. Instructions in How to Posting your Research Abstract

“Start a New Thread” in LEARN (D2L) Course website:

- Under Connect > Discussions > Abstract & Reference Submission > Thread of Questions to Oral Presenters > Click “Start a New Thread”
- Once you start a new thread:
  ○ Under “Enter a subject”:
  ○ enter your Research Topic #: (087) Last Name, First Name: title of your chosen topic (that accurately encapsulates the particular focus of your essay and presentation)

Enter research abstract information into the textbox provided of “New Thread” using the following order of items:

- RESEARCH TOPIC #: (between 001–0180)
- YOUR NAME (Last Name, First Name):
- ABSTRACT TITLE (# words of title): title of your chosen topic (no more than 15 words)
- ABSTRACT BODY (# words of abstract body): abstract body (has to be 500–750 words)
- GENERAL REVIEW ARTICLES FOR REFERENCE (1 or 2): (append reference(s) as attachments in PDF format)
- SPECIFIC ARTICLES FROM HIGH IMPACT FACTOR JOURNALS ILLUSTRATING EXPERIMENTAL RESULTS FOR REFERENCE (3 to 5): append references as attachments in PDF format

## Appendix E. Instructions in How to Create a Narrated Oral Presentation Video in MP4 Format

Key requirements for your oral presentation:

- Your oral presentation must be less than 30 min long.
- Make sure that you export your presentation in a smallest-sized, “Standard (480p)” file format (less than 100 MB).
- Export your oral presentation as an MP4 video.
- Exported MP4 videos can be reprocessed to minimize video file sizes using open-sourced video creation software, such as VideoLAN Movie Creator (VideoLAN).

You can generate your narrated oral presentation by:

- narrating your Slide Show in Powerpoint (PC or MacOS)
- Screen-casting using BONGO Video Assignment Tool within the LEARN (D2L) course website.
- Use free or commercial video creation and editing software (e.g., Camtasia)

If you need extra help in creating a narrated PowerPoint Presentations:

- there are many videos available on YouTube outlining how to create narrated PowerPoint presentations for PC or MacOS.
- contact the IT department computing helpdesk.

Under LEARN (D2L), > Content > download “*First two slides of your oral presentation*” and append these first two slides to your oral presentation before exporting your oral presentation as an MP4 video.

- The first slide is a common first slide for every student, so that every posted oral presentation is anonymous until a peer reviewer opens the oral presentation to view.
- The second slide is an oral presentation identification slide, a template slide for you to enter the:
  ○ Research Topic # from (001 to 181)
  ○ author of the oral presentation (listed as last name, first name)
  ○ oral presentation title
  ○ length of time of the oral presentation.

Warning! Once you upload your oral presentation, it can take up to 2x the video length for the Bongo video assignment tool to process your video before you can submit it.

## Appendix F. Instructions in How to Submit “at Least One Thoughtful and Unique” Question in a “Reply” to “Thread”

After watching the oral presentation video, you will prepare to submit a *thoughtful and unique* question as a “Reply” to the “Thread”. There are many steps that you can take in preparation to submit a *thoughtful and unique* question as a “Reply” to the “Thread”:

- Review the list of previous questions posed by other peer reviewers of the oral presentation topic entered by Reply to “Discussion Thread” in the same or previous weeks.
- Watch video responses to questions posed by the oral presenter in previous weeks.by going to Content > BONGO VIDEO ASSIGNMENTS > *WEEK X VIDEO SUBMISSION OF RESPONSE TO QUESTIONS POSED BY FOUR STUDENTS ON YOUR ORAL PRESENTATION* (where X = 1 to 5) in the LEARN (D2L) course website.
- You might want to continue in a thread of questions based on previous questions posted under the research topic Discussion Thread, and/or questions emerging from having watched video responses to questions posed by the oral presenter in previous weeks.
- I also recommend that you download and read the review manuscripts or scientific manuscripts (in PDF format) posted by the oral presenter under the research topic Discussion Thread. The greater the depth in which you explore the oral presentation topic by watching or reading the available content provided by the oral presenter, the more likely that the oral presenter will grade you positively for having posed a *thoughtful and unique* question under their research topic Discussion Thread.

How to post your *thoughtful and unique* question as “Reply to Discussion Thread”:

- Press “Reply” to research topic “Discussion Thread”
- Before entering your question, type in your research topic identifier (001 to 180) so the oral presenter knows who to evaluate in their quiz evaluation on whether you had posted a *thoughtful and unique* question to them.

Before you complete your peer review feedback questionnaire and student ranking of the four oral presentations which you viewed during the five weeks or oral presentation peer review:

- Go to Content > BONGO VIDEO ASSIGNMENTS > *WEEK X VIDEO SUBMISSION OF RESPONSE TO QUESTIONS POSED BY FOUR STUDENTS ON YOUR ORAL PRESENTATION* (where X = 1 to 5), watch the video response to the question which you posed to the oral presenter

## Appendix G. Instructions in How to Find Your Questions Posted of Your Oral Presentation and How to Create and Upload a Question Response Video

Instructions in how to find your questions posted of your oral presentation:

- Go to folder: Connect > Discussions > Abstract & Reference Submission, Thread of Questions to Oral Presenters, then find your oral presentation in the folder, “Sorted by” Author or Subject.
- Scroll through the Discussion Thread under your research topic, and find where students have posted a series of “Replies” to your “Thread” containing questions posed of your oral presentation at the bottom of the “Thread”.
- Every week you should have four different peer reviewers of your oral presentation who have posted questions under your research topic “Thread”
- If you do not have four different peer reviewers posing questions under your research topic “Thread”, do not worry. Some peer reviewers may be late in posting their questions before the weekly deadline. You can address their question later, such as during the following week when they have posted their question.

Instructions in how to create and upload a question response video:

- If you have peers asking *thoughtful and unique* questions of your research topic oral presentation, you likely will have to do some extra research on your research topic, and possibly have to prepare additional PowerPoint slides, or use some of your existing slides from your oral presentation to narrate a response to questions posed under a “Reply” to your Discussion Thread.
- Follow all the same instructions in *Appendix E—Instructions in how to create a narrated oral presentation video in MP4 format*
- The major difference compared to your uploaded oral presentation video is that you are uploading a single video response addressing the four members of the audience in your virtual classroom, that is questions posed from up to four different questioners of your oral presentation video.
  ○ The second slide of your oral presentation will include the research topic number identifier of the different questioners, and the questions that they posed to you under your Discussion Thread.
  ○ In your narrated PowerPoint presentation, you will first state the research topic number identifier of the questioner and then read aloud the posed question, before answering your response to a questioner. Then you will repeat the process with the next questioner, and so on.
- Submit your MP4 video response to questions under the correct folder: Content > BONGO VIDEO ASSIGNMENTS > *WEEK X VIDEO SUBMISSION OF RESPONSE TO QUESTIONS POSED BY FOUR STUDENTS ON YOUR ORAL PRESENTATION* (where X = 1 to 5).

## Appendix H. Instructions in How to Measure and Evaluate the Quality of Questions of Your Oral Presentation from Questioners

Finding the anonymous quiz for evaluation in the LEARN (D2L) course website:

- Submit > Quizzes > Evaluation of Questioner of Your Oral Presentation > Evaluate the four Questioners of Your Oral Presentation under (XXX) Questioner of Your Oral Presentation, where (XXX) is the questioners research topic identifier.

When responding to whether you were asked a good question from the poser of the question, consider the following:

- Is it a novel question that has not been asked before, which might have been responded to in a previous Video Assignment response?
- Is it a relevant, insightful question that demonstrates that the questioner has watched your oral presentation, watched your previous video responses to questions, and read the content in the abstract and scientific review and experimental manuscripts that were provided under your Discussion Thread?
- Was the question submitted before the deadline on your Discussion Thread?

The quiz is completely anonymous. All quiz scores are collated for a final score after the class term ends.

## Appendix I. Instructions in How to Evaluate Oral Presenters by Feedback Questionnaire Rubric

Finding the anonymous quiz for evaluation in the LEARN (D2L) course website:

- Go to Submit > Quizzes > Evaluation of Oral Presentation > Evaluate the four oral presentations that you reviewed under (XXX) [Title of Oral Presentation]

You will evaluate the oral presentation video and video response to the question(s) posed by you under the “Reply” to Discussion Thread using these four entries:

- (1) Quality of presentation delivery: Choose between *Excellent*—*Very Good*—*Good*—*Satisfactory*—*Fair*
- (2) Quality of presentation materials: Choose between *Excellent*—*Very Good*—*Good*—*Satisfactory*—*Fair*
- (3) Quality of responses to questions answered by posted video assignment: Choose between *Excellent*—*Very Good*—*Good*—*Satisfactory*—*Fair*
- (4) Please provide anonymous constructive comments for the oral presentation which you reviewed (in the textbox provided below).

## Appendix J. Instructions in How to Rank the Four Oral Presentations That You Have Watched and Assessed during the Week

To find the oral presentation ranking quiz:

- Go to Submit > Quizzes > Ranking of Oral Presentations > Rank the four oral presentation which you watched in the week
- Rank the four oral presentation which you watched in the previous week choosing research topics as either in category of *best*, *second best*, *third best,* and *least favorite*.

Notably, you are forced into ranking presentations “*without allowing a tie*” of ones you might think deserving of equal merit:

- With everyone having to do the same ranking evaluation, it balances out with a large sample size of 20 reviewers of each presentation from a review pool of maximum 180.
- Statistically-speaking, others will also find some oral presentations difficult to rank, and will choose an opposite scoring result.
- A forced ranking scoring system ensures that everyone ranks and compares their four oral presentations that they observe every week.
- If I gave students a choice in ranking oral presentations, they would rank every oral presentation in the single category of *best*.

The ranking scores will provide a final tally from 20 reviewers for each student, and then the scoring rank will be converted to a percentage, where the average student gets in the 70s, and the top student(s) gets 100%:

- The oral presentation section of the course is evaluated by all students participating in the course (and is worth 35% of your final grade).
- The ranking quizzes are completely anonymous and will be collated for final marks after the class term is over.
- Participation marks are provided for those who complete the instructed tasks on time (20% of final grade).
- All written assessments (45% of the final grade) are marked by the instructor.

## Appendix K. Instructions in How to Draft Your Final Written Submissions (Serving in lieu of a Final Exam) and How They Will Be Assessed

Instructions in how to draft your final Research report:

- Your written research report is worth 15% of your final grade, and due by the end of the final exam period.
- Your written research report will be in a Letters to Nature format. View link: https://www.nature.com/nature/for-authors/formatting-guide (accessed on 24 May 2021)
- Letters to Nature are short reports of original research focused on an finding whose importance means that it will be of interest to scientists in other fields.
- Letters to Nature formatted essays begin with a fully referenced *summary paragraph*, aimed at readers in other disciplines.
- This paragraph starts with a basic introduction to the field in 2 to 3 sentences, followed by a one-sentence statement of the main conclusions starting with ‘Here we show’ or an equivalent phrase; finally, add 2–3 sentences putting the main findings into a general context so it is clear how the results described in the paper have moved the field forward.
- Please refer to the example to see how the *summary paragraph* for a Letter should be constructed. View link: https://www.nature.com/documents/nature-summary-paragraph.pdf (accessed on 24 May 2021)
- Any discussion at the end of the text should be as succinct as possible, to briefly convey the general relevance of the work.
- The nature summary paragraph is not an abstract should have a maximum of 150 words, summarizing the background, rationale, main results, and implications.
- Main body text of a Letters to Nature is limited to 2000 words, excluding the Summary paragraph, Methods, reference sections, and figure legends.
- Letters should have no more than 3–5 display items (figures and/or tables)

Instructions in how to draft your final Research essay:

- Your written research report is worth 15% of your final grade, and due by the end of the final exam period.
- You will write a research essay on one of the 180 research topics in the course, given to you at random by Week 6 of the course.
- The purpose of the research essay is research and written communication practice on a systems neuroscience topic, but where you do not get to choose your topic. Everyone obtains a different essay topic.
- You will learn a lot through the process, and should find the study of the topic interesting.
- You learn more and enjoy the process more in a self-guided investigation of a topic where you sift through different information, rather than being told what is the truth provided in details that you memorize from PowerPoint slides or from a textbook.
- The research essay is to be a maximum of 2500 words, which is approximately 10 pages. You are to provide at least 10 references.
- I am looking for your depth of knowledge in the detailed, molecular, cellular, network, computational, and behavioral aspects relevant to each systems neuroscience topic.
- If it is about a particular neural process, you must go into details of the neural network wiring underlying the behavior.
- You do not have to cover every aspect of the research topic.
- I am looking at a measure of the depth at which you have investigated your topic.
- Organize your ideas under topic subheadings of your choosing. Do not format the Research Essay like the Research Report with subheadings of Introduction—Methods –Results—Discussion—Conclusion, and Future Outlook.
- It is important to make reference to particular experimental methods and outcomes to support your ideas in the essay.
- Unlike the research report, you do not embed figures from experimental scientific manuscripts in this research essay.
- I can tell which of the students have read a lot in preparation for their essay. Because you include citations, and you submit your essay to Dropbox where it is evaluated with Turnitin software, I can follow the references where you get your information, and how broadly and deeply you have investigated your topic.
- I learn a lot just by reading your essay submissions and will be particularly pleased when a student has gone beyond what I know on any of the topics

How your Research report and Research essay will be assessed:

Submit your written work to: Submit > “Dropbox” to appropriate folder, all which are linked to text matching assessment software, Turnitin^®^ used for screen assignments for plagiarism *****:

- (A) Abstract and References submission (*due by Week 6*)
- (B) Research Report on your chosen neuroscience topic (*due by end of the final exam period*)
- (C) Research Essay on assigned neuroscience topic (*due by end of the final exam period*)

Your submitted written work: abstract, essay, report (each worth 15% of the final grade) will be evaluated in three categories:

- (1) Abstract/essay content and structure for developing research topic argument (1/3 of mark)
- (2) Evidence in scientific references for supporting hypothesis/questions addressed in research topic (1/3 of mark)
- (3) Clarity, grammar, style of written essay (1/3 of mark)

For the three categories above, you will be given a score from 1 to 5 based on the following rubric (5 = *excellent*, 4 = *very good*, 3 = *good*, 2 = *satisfactory*, 1 = *fair*).

Late essays lose 1/2 of final grade for essay component (7.5%)

*Plagiarism of written work:

It is not just about “copying and pasting” text from another source: Plagiarism is defined as the taking of another author’s “language, thoughts, ideas, or expressions” and the representation of them as one’s own original work.

- To avoid detection of plagiarism by Turnitin software, students will copy over a text from a source, then change a few words or the way the sentence or paragraph or essay is organized. The primary issue with plagiarism is not just the simple “copying” and “pasting” of the sentence or paragraph structure from a source and calling it your own.
- The primary issue with regards to plagiarism is not in word or sentence choices, it is in the taking of ideas as a single author has conceived them.
- I do not want you to take a single author’s opinion as gospel. You are to create a “novel” piece of work, that has never been created before.
- Students will say that they cannot avoid using certain specific language in the technical sciences, like systems neuroscience, because certain phrases are repeated because it is simpler to use particular technical jargon than to avoid the technical jargon.
- You should avoid technical jargon when you can, because it is often only understandable by those that use the particular phrasing in a subfield.
- You should write as much as possible with simple, clear language that is as accessible to others as possible.
- You do not write to impress your audience with the complexity of your writing. The best writing is simple and clear and accessible to anyone, including those whom are not educated in the sciences.

If you read broadly and deeply then you will begin to have your “own voice” on the research topic:

- I want you to read from many different sources, including both review publications and experimental papers.
- If you have read enough, you will not need to copy someone’s text. You will begin to have your “own voice” on the topic.
- As you read many review and experimental manuscripts, you will become familiar with the consensus “*accepted*” position of the field that is repeated as a starting point in the introduction for every manuscript.
- You will be more confident in your opinion on the research topic, because you will have read both broadly and deeply on your topic.
- Every subfield has many different researchers, with varying approaches and opinions, and often there can be completely different, but completely accepted views of how we believe things work on a topic of Systems Neuroscience.
- What we believe to be true is limited by the experimental results found based on a particular experimental design, which can provide windows of opportunity for understanding of a mechanism, but most experimental designs also have weaknesses.
- Often there can be completely opposing interpretations and conclusions of the same set of experimental results.
- You are going to have to defend your research topic in an oral (and possibly poster) presentation by questioners, so you must have a depth of understanding of your topic, beyond just a single author’s opinion on a topic.
- If you read broadly and deeply enough, you will begin to be able to think and write on your research topic more easily, and you will naturally avoid “copying and pasting” a single author’s work in your research abstract or report.

## Appendix M. Sample Student Feedback Comments for the Course

Positive student feedback on delivery of oral/written communication course by asynchronous, remote learning delivery method:

*“I think this course suits an online format quite well actually. Allowing students to watch presentations on their own time and coming up with questions suited an online format better than an in-person format in my opinion. I find it difficult to come up with questions on the spot sometimes when watching a live presentation, and so being able to rewind and re-listen to presentations allowed me to come up with more detailed and thoughtful questions than I would have been able to in standard in-person presentations.”*

*“I really like the independent, self-directed mode of learning in this course, it is extremely different from any other course I have taken. It takes a lot of time management and self-discipline to be able to regulate your work with very few strict deadlines. One of my favourite things about this class was that it provided me with the opportunity to practice my scientific writing. Few courses provide students with opportunities like this to write these kinds of scientific papers while requiring students to do the research completely on their own. I feel as though my writing has improved and I am much more comfortable with the writing process than I was before.”*

*“Overall, this course was different from the usual course which makes it memorable and quite interesting. I found that I learned a lot from my own topic and those of my peers.”*

*“Loved the course and had a lot of fun learning about something in-depth. Peripheral knowledge and simple facts are sooo overrated.”*

*“When I started off this course, I didn’t realize how much fun it would be doing the presentation and research. I found this to be one of the most interesting courses till now. And I really liked the course format.”*

*“I enjoyed learning about the different neuroscience topics through research studies compared to the more traditional lecture-based learning. Through my research, I was able to learn about my topic from different cellular, molecular, and behavioural perspectives which I believe helped me really understand my topic. Learning about past, present, and future research approaches was also really interesting. I really enjoyed the presentations that related neuroscience topics to human diseases.”*

*“Yes, being able to watch other presentations and review them has taught me a lot about what makes a good presentation. I have learned about the different ways in which the information related to our topics can be presented so that other students are able to understand the material. The feedback I have received from other students has allowed me to identify areas that I can improve in in order to be a better presenter. Overall, I think the course has taught me a lot of skills related to presenting and doing research that I don’t think I would have been able to learn in other courses.”*

*“I think of all the classes I’ve taken so far, this is easily my favourite, and I really enjoyed the challenge of the class, while still finding the course very engaging and interesting. The teaching style was unique, but worked well for me, and allowed me to ask questions of other presenters, that I personally found interesting, like anti-aging or cancer, and its relation to neuroscience. This class helped me realize I want to focus my studies in one of these fields after my undergrad and gave me a great passion for them. Having the final be replaced with two papers, tested my knowledge more than any exam could, and was a more enjoyable alternative, that let me practice my scientific writing skills. Overall, the personalized learning style of the course works tremendously, and I’d recommend this course to any 4th year student.”*

*“I genuinely learnt a lot in terms of research, how to read and go through journal articles, and be able to synthesize information.”*

*“The course is really nice, a good nice change from other classes.”*

*“I enjoyed this course and enjoyed having the opportunity to actively engage with neuroscience research.”*

*“I genuinely learnt a lot in terms of research, how to read and go through journal articles, and be able to synthesize information.”*

Negative student feedback on delivery of oral/written communication course by an asynchronous, remote learning delivery method:

*“Personally, I feel that given my learning style, I would have preferred more feedback as I benefit most from in person interaction and discussion.”*

*“It felt disorganized during the 5-week peer evaluation period because the discussion board was a mess of threads. Same with the order/structure of the quizzes. Additionally, it’s unclear how the ranking system will be reflected in our grade.”*

*“Maybe give more structure on teaching people HOW to teach. A lot of people had cool topics but they just were all over the place, no flow”*

*“Some presentations mainly focused on the experiments, and less on the neurological background science, and a small shift to focus the importance of the background information would be helpful.”*

*“I am not sure if the ranking evaluation is fair for the 35% of the mark. Although rankings are random, the question poses what if 4 very great presentations were given? In the end, will is equal out?”*

*“It would have been nice to be given the chance to select for a topic to learn more about, rather than an auto-generation of videos that we have to watch.”*

Negative student feedback on use of the BONGO Video Assessment Tool:

*“BONGO was a pain and should not be used again in the future. It messes up with the audio of some videos and it has a ridiculous long upload time. Overall, BONGO was not a good choice for this course.”*

*“I would highly recommend this course if BONGO was not used. I experiences far too many technical difficulties with BONGO that at this point I am not sure how it out of the programming phase. They need to seriously work on this software. The tech support team offered was lovely and I am not trying to offend them at all. My issue relies solely with BONGO. A complete nightmare of a software. Please seriously consider not using BONGO for future online courses. Trust me your student’s morale will go through the roof.”*

*“BONGO was a not a good tool to use for this class. It happens to always be malfunctioning in some way. For some people, my video would be just fine, whereas for others, the audio would be cut off. The Submission List for amount of peer videos you’ve watched is also a little confusing. The first video that we’ve watched from our peers would be the Submission with the highest number (i.e., in week 3, we would have watched 12 videos and the 12th video on our submission list is the first video that we watched from week 1). The processing time for a video to be uploaded is also sometimes absurd, it could range from a few minutes to a day.”*

Negative student feedback on the time commitment required to complete the course:

*“It’s definitely the course that took up most of my time this semester. September to October 13th, it was about par with my other courses (I would spend roughly 20 h a week reading articles about my topic, summarizing these articles, preparing my abstract and presentation). However, once we transitioned into the second phase (video reviews, rankings, questions --> 35%) I found that it now became the course that took up the majority of my time. This is a rough estimate but I’m taking 5 courses this term, and I found that this course in particular took about 50% of my study time (instead of 20% - if each class was divided equally). I would spend roughly two days to view the 4 ~25-min videos, read corresponding articles and abstract and about 2 days to research and prepare a ~15-min presentation answering the questions I received. I found that I now usually had only three days in the week to work on my other 4 classes. With that said, it was a very unique course. I learned so much about my chosen and assigned neuroscience topics. Above the conventional science, I also learned how to interpret published articles (which I have struggled with in the past), make sense of the important information, and summarize other findings. No other course would have given me the opportunity to develop these important skills, so thank you! Another new experience I gained from this course was the ability to receive and give feedback to my peers. I wonder if everyone took the task of watching 4 presenters and formulating thoughtful questions as serious as I did, but I would hope so (sometimes, the questions I received (although not bad enough to warrant a ““did not ask a thoughtful question) did not show much interest/engagement in the presentation). I did learn a lot about my presentation style and have lots to improve for future courses/career. Some big take a ways for me are: my slides are too text heavy, I speak too fast, my slides have too much colour. I’ll work on these aspects in the future. Last thing I wanted to say is, I had mixed feelings about this course until I finished the essay and report. Only after submitting these final assessments did I realize just how much I learned.*

*“I did feel that this course was on the heavy side and maybe watching 4 whole videos every week was excessive, especially when you had to come up with a question, fill out 3 quizzes, and come up with a response video all in the same week. I loved it, but I felt like I would have gotten the same experience if I had to only watch 3 videos lets say”*

*“too much week-to-week evaluation required of presentations (~2hrs of lecture plus all the quizzes) and 2 separate essays at the end of the course, both with confined word limits (2000wds), was a bit excessive to me”*

*“~5 weeks given for us to research the topic and give an abstract and presentation was well done. In terms of evaluation, having to do 4 videos each week followed by doing a presentation each week, I found to consume a lot of time.”*

*“While I expect a 4th year course to have a heavy workload, the workload of this course was at times excessive. Especially during the second portion of the course, having to watch about 2 h of presentations a week, and then come up with questions, which as the term progressed took longer and longer because the most obvious questions had been asked, and then do additional research of the questions asked of your own presentation, and create and record a new slide show, I was investing anywhere between 8–12 h a week in this course alone, compared with 4–6 h for most of my other courses.”*

*“In addition, the 2nd portion of the course is a bit time consuming, perhaps creating video responses can be in the span of 2 weeks instead of every week? The current format is manageable, however, approaching Week 4, creating unique and thoughtful questions is a bit more difficult.”*

*“I did find the course to be very heavy on the workload at times, especially during this second portion of the course. There is a lot of time spent watching presentations and then coming up with good questions often requires more digging or rewatching parts of the presentation. And then answering other people’s questions also takes several hours as I often have to conduct additional research to provide a quality answer. While I would expect a 4th year course to have a heavy workload, I think it is also exacerbated a little in this course because the five-week review period provides no breaks, so if you get a little behind one week because of midterms or other assignments, it is very difficult to get back on track for the next weeks.”*

*“It was hard to balance the workload at times, particularly in the last half of the course when reviewing of oral presentations and answering questions started. I struggle with feeling as though I have done enough or adequate research for my topics as feedback doesn’t come until later in the course.”*

## References

[1] Parker S.W., Hansen M.A., Bernadowski C. (2021). COVID-19 Campus Closures in the United States: American Student Perceptions of Forced Transition to Remote Learning. Soc. Sci..

[2] Meulenbroeks R. (2020). Suddenly fully online: A case study of a blended university course moving online during the Covid-19 pandemic. Heliyon.

[3] World Health Organization (2020). Considerations for School-Related Public Health Measures in the Context of COVID-19: Annex to Considerations in Adjusting Public Health and Social Measures in the Context of COVID-19, 14 September 2020 (No. WHO/2019-nCoV/Adjusting_PH_Measures/Schools/2020.2).

[4] Al Samaraee A. (2020). The impact of the COVID-19 pandemic on medical education. Br. J. Hosp. Med..

[5] Kim J.W., Myung S.J., Yoon H.B., Moon S.H., Ryu H., Yim J.-J. (2020). How medical education survives and evolves during COVID-19: Our experience and future direction. PLoS ONE.

[6] Alsoufi A., Alsuyihili A., Msherghi A., Elhadi A., Atiyah H., Ashini A., Ashwieb A., Ghula M., Ben Hasan H., Abudabuos S. (2020). Impact of the COVID-19 pandemic on medical education: Medical students’ knowledge, attitudes, and practices regarding electronic learning. PLoS ONE.

[7] Tolsgaard M.G., Cleland J., Wilkinson T., Ellaway R.H. (2020). How we make choices and sacrifices in medical education during the COVID-19 pandemic. Med. Teach..

[8] Ramos R.L. (2020). When the COVID-19 Pandemic Changed Neuroscience Education. J. Undergrad. Neurosci. Educ..

[9] Ramos R.L. (2020). Virtual was the Reality in Neuroscience Education during the COVID-19 Pandemic. J. Undergrad. Neurosci. Educ..

[10] Lillejord S., Børte K., Nesje K., Ruud E. (2018). Learning and teaching with technology in higher education-a systematic review. Oslo: Knowl. Center Educ..

[11] Cheung S.K.S., Kwok L.F., Phusavat K., Yang H.H. (2021). Shaping the future learning environments with smart elements: Challenges and opportunities. Int. J. Educ. Technol. High. Educ..

[12] Sweetman D.S. (2021). Making virtual learning engaging and interactive. FASEB BioAdvances.

[13] Dumford A.D., Miller A.L. (2018). Online learning in higher education: Exploring advantages and disadvantages for engagement. J. Comput. High. Educ..

[14] Bordes S.J., Walker D., Modica L.J., Buckland J., Sobering A.K. (2021). Towards the optimal use of video recordings to support the flipped classroom in medical school basic sciences education. Med. Educ. Online.

[15] Hew K.F., Lo C.K. (2018). Flipped classroom improves student learning in health professions education: A meta-analysis. BMC Med. Educ..

[16] Ramnanan C.J., Pound L.D. (2017). Advances in medical education and practice: Student perceptions of the flipped classroom. Adv. Med. Educ. Pr..

[17] Clark C., Strudler N., Grove K. (2015). Comparing asynchronous and synchronous video vs. text based discussions in an online teacher education course. Online Learn..

[18] Thomas R.A., West R.E., Borup J. (2017). An analysis of instructor social presence in online text and asynchronous video feedback comments. Internet High. Educ..

[19] Choe R.C., Scuric Z., Eshkol E., Cruser S., Arndt A., Cox R., Toma S.P., Shapiro C., Levis-Fitzgerald M., Barnes G. (2019). Student Satisfaction and Learning Outcomes in Asynchronous Online Lecture Videos. CBE—Life Sci. Educ..

[20] Dennen V.P. (2005). From message posting to learning dialogues: Factors affecting learner participation in asynchronous discussion. Distance Educ..

[21] Namin A., Ketron S.C., Kaltcheva V.D., Winsor R.D. (2021). Improving Student Presentation Skills Using Asynchronous Video-Based Projects. J. Manag. Educ..

[22] Schultz P.L., Quinn A.S. (2013). Lights, Camera, Action! Learning About Management With Student-Produced Video Assignments. J. Manag. Educ..

[23] Prud’Homme-Genereux A. (2016). Case Study: Student-Produced Videos for the Flipped Classroom. J. Coll. Sci. Teach..

[24] Annan K., Onodipe G., Stephenson A. (2019). Using Student-Created Content Videos in Flipped Learning to Enhance Student Higher-Order Thinking Skills, Engagement, and Satisfaction. J. Educ. Soc. Policy.

